# IPE CASE STUDY ON COMMUNICATION AND ASSESSMENT OF OLDER ADULTS: A COMPARISON OF THREE ONLINE MODALITIES

**DOI:** 10.1093/geroni/igae098.0051

**Published:** 2024-12-31

**Authors:** Tara Sharpp, Laura Gaeta

**Affiliations:** California State University Sacramento, Sacramento, California, United States; California State University Sacramento, California State University, Sacramento, California, United States

## Abstract

Interprofessional education (IPE) is critical for health profession students to learn teamwork, shared experiences, and communication. In this mixed-method study, we compared an IPE activity for Nursing, Audiology, Communication Sciences, Gerontology, and Health Science students using three online modalities: (1) Synchronous online, (2) Asynchronous online, and (3) Bichronous online (combination of asynchronous and synchronous). In IPE groups, the students examined the same unfolding case study about an older adult with hearing loss, depression, and dementia. Over three terms, 168 students completed pretest and posttest measures, including a standardized IPE scale (IPAS subscale), a knowledge quiz, and an evaluation. Knowledge quiz scores significantly improved in all three groups (synchronous: t = -2.586, 2-sided p = 0.013; asynchronous: t = -4.129, 2-sided p < 0.001; bichronous: t = -3.748, 2-sided p < 0.001). Students also demonstrated significantly increased attitudes toward working in teams in the IPAS subscale in the posttest in all three groups. ANOVA and Kruskal Wallis tests demonstrated no statistically significant differences in the IPAS scale and evaluation scores between groups, indicating that students in all three modalities had similar levels of learning regarding their IPE. In the open-ended responses of the evaluation, students reported they learned practical examples of how to work with team members from their group and that they valued the online experience. The results indicate that health professions students can effectively learn from a case study activity how to communicate with older adults and work in interprofessional teams in various forms of online modalities.

